# Strong Correlations between the Binding Antibodies against Wild-Type and Neutralizing Antibodies against Omicron BA.1 and BA.2 Variants of SARS-CoV-2 in Individuals Following Booster (Third-Dose) Vaccination

**DOI:** 10.3390/diagnostics12081781

**Published:** 2022-07-22

**Authors:** Nungruthai Suntronwong, Suvichada Assawakosri, Sitthichai Kanokudom, Ritthideach Yorsaeng, Chompoonut Auphimai, Thanunrat Thongmee, Preeyaporn Vichaiwattana, Thaneeya Duangchinda, Warangkana Chantima, Pattarakul Pakchotanon, Jira Chansaenroj, Pornjarim Nilyanimit, Donchida Srimuan, Thaksaporn Thatsanatorn, Natthinee Sudhinaraset, Nasamon Wanlapakorn, Juthathip Mongkolsapaya, Yong Poovorawan

**Affiliations:** 1 Center of Excellence in Clinical Virology, Faculty of Medicine, Chulalongkorn University, Bangkok 10330, Thailand; suntronwong.n@gmail.com (N.S.); suvichada.assawa@gmail.com (S.A.); kanokudom_s@yahoo.com (S.K.); ritthideach.yor@gmail.com (R.Y.); chompoonut.bit@gmail.com (C.A.); tata033@hotmail.com (T.T.); preeya_teiy@hotmail.com (P.V.); job151@hotmail.com (J.C.); mim_bhni@hotmail.com (P.N.); donchida.s@gmail.com (D.S.); thaksapohnl@hotmail.com (T.T.); dr_natthinee@hotmail.com (N.S.); nasamon.w@chula.ac.th (N.W.); 2 Molecular Biology of Dengue and Flaviviruses Research Team, National Center for Genetic Engineering and Biotechnology (BIOTEC), National Science and Development Agency, NSTDA, Pathum Thani 12120, Thailand; thaneeya.dua@biotec.or.th (T.D.); mk_pk@msn.com (P.P.); 3 Division of Dengue Hemorrhagic Fever Research, Faculty of Medicine Siriraj Hospital, Mahidol University, Bangkok 10700, Thailand; warangkana_ch1@hotmail.com; 4 Siriraj Center of Research Excellence in Dengue and Emerging Pathogens, Faculty of Medicine Siriraj Hospital, Mahidol University, Bangkok 10700, Thailand; 5 Wellcome Centre for Human Genetics, Nuffield Department of Medicine, University of Oxford, Oxford OX3 7BN, UK; jmongkol@well.ox.ac.uk; 6 Chinese Academy of Medical Science (CAMS) Oxford Institute (COI), University of Oxford, Oxford OX3 7BN, UK; 7 The Royal Society of Thailand (FRS(T)), Sanam Sueapa, Dusit, Bangkok 10330, Thailand

**Keywords:** COVID-19, SARS-CoV-2, neutralization, omicron, antibody, correlation

## Abstract

This study examined the neutralizing activity and receptor-binding domain (RBD) antibody levels against wild-type and omicron BA.1 and BA.2 variants in individuals who received three doses of COVID-19 vaccination. The relationship between the anti-RBD IgG against wild-type and live virus neutralizing antibody titers against omicron BA.1 and BA.2 variants was examined. In total, 310 sera samples from individuals after booster vaccination (third-dose) were tested for specific IgG wild-type SARS-CoV-2 RBD and the omicron BA.1 surrogate virus neutralization test (sVNT). The live virus neutralization assay against omicron BA.1 and BA.2 was performed using the foci-reduction neutralization test (FRNT50). The anti-RBD IgG strongly correlated with FRNT50 titers against BA.1 and BA.2. Non-linear regression showed that anti-RBD IgG at the cut-off value ≥148 BAU/mL and ≥138 BAU/mL were related to the threshold for FRNT50 titers ≥20 against BA.1 and BA.2, respectively. A moderate correlation was observed between the sVNT and FRNT50 titers. At FRNT50 titers ≥20, the predicted sVNT for BA.1 and BA.2 was ≥10.57% and ≥11.52%, respectively. The study identified anti-RBD IgG and sVNT levels that predict detectable neutralizing antibodies against omicron variants. Assessment and monitoring of protective immunity support vaccine policies and will help identify optimal timing for booster vaccination.

## 1. Introduction

Detection of neutralizing antibodies helps to predict humoral immunity protection and monitor waning immunity and vaccine immunogenicity. Numerous studies have shown that a high level of neutralizing antibodies is correlated with SARS-CoV-2 protection and reduces the severity of the disease [1,2,3]. However, the current gold standard neutralization assay (live virus neutralization assay) has been limited for widespread use due to the need for specially trained personnel to handle the live SARS-CoV-2 virus and the need to work in a biosafety level 3 laboratory (BSL3) containment facility. 

Although several commercial kits were available to detect the SARS-CoV-2 antibody and are currently used in hospital and clinical laboratories, the antigens tested derived from the ancestral strain because tests were developed before the SARS-CoV-2 variants emerged [4]. Furthermore, surrogate virus neutralization (sVNT) is widely performed to determine the ability to neutralize antibodies to block the interaction between the receptor-binding domain (RBD) and human ACE2 receptors [5]. Several studies have shown that the commercial binding antibody assay and sVNT are well correlated with the gold standard results of the neutralization method against the ancestral strain [6,7,8]. Due to the spread of SARS-CoV-2 omicron variants, concerns have been raised as to whether preexisting immunity is sufficient to prevent omicron infection. However, the relationship between the anti-RBD IgG against wild-type and live virus neutralization assay against omicron has been limited.

In this study, we applied non-linear regression analysis to predict the level of anti-RBD IgG and sVNT related to the detectable level of FRNT50 titers against omicron variants, including the BA.1 and BA.2 subvariants, in serum collected from individuals after receiving the COVID-19 booster (third-dose) vaccination.

## 2. Materials and Methods

### 2.1. Participants and Ethical Considerations

Our study recruited 310 sera samples from individuals after receiving the booster (third-dose) COVID-19 vaccination from previous studies [9,10]. There were two primed cohorts for analysis. The first cohort was primed with two doses of AZD1222 and boosted with AZD1222, BNT162b2, 50 µg of mRNA-1273 or 100 µg of mRNA-1273 6 months after the first vaccination. The second cohort was primed with heterologous CoronaVac/AZD1222 and boosted with AZD1222, BNT162b2, and 100 µg mRNA-1273 approximately 4–5 months after the initial vaccination. The enrollment period was between November 2021 and January 2022. Blood samples were collected at day 0 and at day 28 in all vaccine regimens, while blood samples at day 90 post-booster were collected only from individuals boosted with BNT162b2 or 50 µg or 100 µg of mRNA-1273 following two doses of AZD1222. This study was performed following the Declaration of Helsinki and Good Clinical Practice principles. The study protocol was reviewed and approved by the Institutional Review Board of the Faculty of Medicine of Chulalongkorn University (IRB numbers 871/64 and 690/64). All participants signed a written consent form before being enrolled.

### 2.2. Measurement Anti-RBD IgG and sVNT 

All sera samples were quantitatively measured for wild-type SARS-CoV-2 receptor-binding domain (RBD) specific IgG (anti-RBD IgG) using the commercial assay, Abbott SARS-CoV-2 IgG II Quant assay (Abbott Diagnostics, Abbott Park, IL, USA). Anti-RBD IgG was reported as a binding antibody unit (BAU/mL). The surrogate virus neutralization assay (sVNT) against variants of BA.1 was performed using a cPassTM SAR-CoV-2 neutralizing antibody detection kit (GenScript Biotech, Piscataway, NJ, USA) as previously described [9,10].

### 2.3. Foci-Reduction Neutralization Test (FRNT50)

For the live virus neutralization test, the foci-reduction neutralization test (FRNT50) was performed using the live SARS-CoV-2 virus, which included the omicron BA.1 (GISAID accession number: EPI_ISL_8547017) and BA.2 (accession number: EPI_ISL_11698090) subvariants as previously described [9,10]. The cut-off level of FRNT50 titer ≥20 was considered a detectable level of neutralizing antibody. If the neutralizing antibody titer was undetected (the cut-off value of FRNT50 titer <20), the FRNT50 was set as 10.

### 2.4. Statistical Analysis

For statistical analysis, the predicted values of anti-RBD IgG and the percentage of inhibition measured by sVNT at the cut-off value for FRNT50 titers ≥20 and ≥40 were determined using non-linear regression analysis and performed on the log10 transformed data. The Spearman’s rank correlation between anti-RBD IgG, the percentage of inhibition measured by sVNT, and FRNT50 titers was determined using SPSS v23.0 (IBM Corp, Armonk, NY, USA). The r-square was calculated according to the non-linear equation using STATA v.17.0 software. A *p*-value < 0.05 was considered statistically significant.

## 3. Results

### 3.1. Correlations between Anti-RBD IgG against Wild-Type and FRNT50 Titers against Omicron

**Figure 1:**
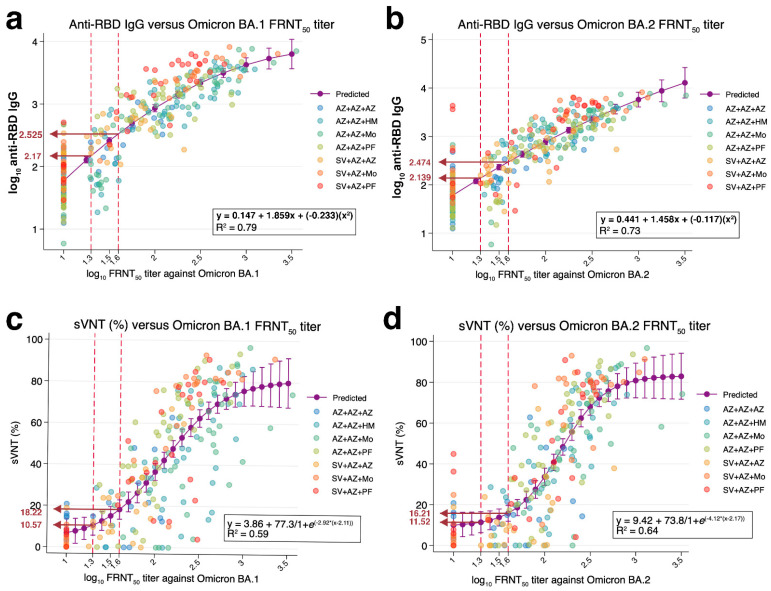
Prediction of the level of anti-RBD IgG tested against the ancestral strain and the percentage of blocking between RBD and ACE-2 interaction against omicron based on the FRNT50 assay against SARS-CoV-2 omicron BA.1 and BA.2 variants using non-linear regression analysis. Sera samples from individuals with booster vaccination were tested with anti-RBD IgG against wild-type, sVNT against omicron, and FRNT50 against omicron BA.1 and BA.2. Predicted anti-RBD IgG level (*n* = 310) was based on the FRNT50 titer against BA.1 (Panel (**a**)) and BA.2 (Panel (**b**)). Predicted sVNT level (*n* = 218) was based on the FRNT50 against BA.1 (Panel (**c**)) and BA.2 (Panel (**d**)). The y axis represents log10 scale of anti-RBD IgG (BAU/mL). The x axis represents the log10 of FRNT50 titers. Dotted lines indicate 1.3 (the cut-off value of FRNT50 titer = 20) and 1.6 (the cut-off value of FRNT50 titer = 40). The arrows indicate the predicted level of anti-RBD IgG and percentage of inhibition from sVNT. Colored circles indicate the vaccine regimens for primary vaccine series+ booster vaccine. The r-square was calculated according to the non-linear equation using STATA v.17.0 software. SV = CoronaVac (Sinovac, Beijing, China), AZ = AZD1222 (AstraZeneca, Oxford, UK), PF = BNT162b2 (Pfizer-BioNTech, Mainz, Germany), Mo = full dose mRNA-1273 (100 µg) (Moderna, Massachusetts, MA, USA), HM = half dose mRNA-1273 (50 µg).

A total of 310 sera samples from individuals receiving different booster vaccinations were tested for wild-type anti-RBD IgG and FRNT50 of BA.1 and BA.2 (Table 1). The FRNT50 titer ranged from undetectable (<20) to 3552 for BA.1 and undetectable to 3249 for BA.2 as examined on day 0 and on days 28 and 90. The correlation analysis indicated that anti-RBD IgG was strongly correlated with FRNT50 titers against BA.1 (Spearman’s R: 0.89, *p* < 0.001) and BA.2 (Spearman’s R: 0.86, *p* < 0.001) (Table S1). Non-linear regression analysis showed the predicted anti-RBD IgG was 148 BAU/mL and 335 BAU/mL when the FRNT50 titers against omicron BA.1 were 20 (1.3 of log10 FRNT50 titers) and 40 (1.6 of log10 F FRNT50 titers), respectively (r^2^ = 0.79, *p* < 0.001) (Figure 1a). In addition, the predicted anti-RBD IgG was approximately 138 BAU/mL and 298 BAU/mL when the FRNT50 titer against omicron BA.2 was 20 and 40, respectively (r^2^ = 0.73, *p* < 0.001) (Figure 1b). When the cut-off value of 1:20 for the neutralization test was used for BA.1, the anti-RBD IgG cut-off of 148 BAU/mL showed 89.7% sensitivity and 81.4% specificity, whereas, for BA.2, the anti-RBD IgG cut-off of 138 BAU/mL showed 86.8% sensitivity and 82.9% specificity. In addition, a similar trend of positive correlation between anti-RBD IgG and FRNT50 titers against omicron BA.1 and BA.2 was observed when analyzed by sex (male and female) and age (individuals aged <60 and ≥60 years old) groups (Supplementary Figures S1 and S2).

### 3.2. Correlations between sVNT against Omicron and FRNT50 Titers against Omicron

**Figure 2:**
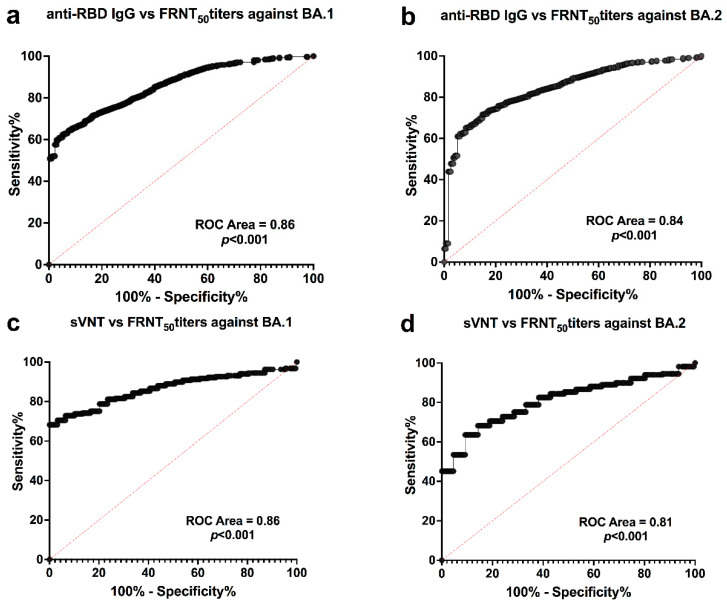
ROC analysis between anti-RBD IgG, surrogate virus neutralization test (sVNT), and FRNT50 titers against omicron BA.1 and BA.2. ROC analyses for anti-RBD IgG tested at cut-off value FRNT50 titer = 20 against BA.1 (Panel (**a**)) and BA.2 (Panel (**b**)). ROC curve of sVNT tested at cut-off value FRNT50 titer = 20 against BA.1 (Panel (**c**)) and BA.2 (Panel (**d**)).

The relationship between sVNT and FRNT50 titers (n = 218) was determined, and a moderate correlation between the sVNT and FRNT50 titers was observed (Spearman’s R = 0.77 and 0.79 for BA.1 and BA.2, *p* < 0.001) (Table S1). Non-linear regression showed that the percentage inhibition of ACE-2 and RBD binding measured by sVNT was 10.57% and 18.22% and was related to the cut-off value of 20 and 40 FRNT50 titers for BA.1 (r^2^ = 0.59, *p* < 0.001) (Figure 1c). However, sVNT was 11.52% and 16.21% and was related to the cut-off value of 20 and 40 FRNT50 titers for BA.2 (r^2^ = 0.64, *p* < 0.001) (Figure 1d). When the cut-off level of 1:20 for the neutralization test was used for BA.1, the sVNT of ≥10.57% showed 86.4% sensitivity and 73.1% specificity, whereas for BA.2, the sVNT of ≥ 11.52% showed 82.3% sensitivity and 63.2% specificity. The ROC analysis indicated that anti-RBD IgG and sVNT provided good performance in detecting neutralizing antibodies against omicron variants (Figure 2). There is no difference observed in the trends of correlation analysis between sVNT and FRNT50 titers against omicron BA.1 and BA.2 analyzed controlling for the groups of sex and age (Supplementary Figures S3 and S4).

## 4. Discussion

Numerous studies have shown a strong correlation between the levels of antibody binding response, including anti-spike, anti-RBD antibodies, and neutralizing antibody titers against ancestral strain in individuals with previous COVID-19 infection or vaccination [6,11,12,13,14]. However, there is evidence that the binding antibody was poorly correlated with neutralizing antibody titers against variants derived from B.1.1.7 and B.1.351 compared to the ancestral strain [7,15]. In this study, we found that the anti-RBD IgG and sVNT tested by commercial kits correlated well with neutralizing antibody titers against the SARS-CoV-2 omicron variants. In addition, our data predicted anti-RBD IgG and sVNT at a detectable level of neutralizing antibodies against omicron BA.1 and BA.2 (FRNT50 titers ≥ 20). These findings suggest that boosting immunity against vaccine strain (ancestral strain) could induce cross-reactivity against omicron variants.

Anti-RBD IgG measured all antibodies targeting receptor binding sites, neutralizing antibodies, and non-neutralizing antibodies. The antigen for anti-RBD IgG detection was designed on the basis of the ancestral strain. Although more than 30 amino acid mutations were detected in the omicron variant spike protein [16], our results show that the anti-RBD IgG and neutralizing antibody tested by FRNT50 titers against the omicron variant provided a strong correlation, which is consistent with a previous report [17]. In addition, correlations between neutralizing activity against variants of SARS-CoV-2 and RBD-specific binding antibody have been reported in samples with high binding antibody titers [7].

However, our result is inconsistent with a previous study [18], which showed that anti-RBD IgG was not correlated with sVNT against omicron variants. The inconsistent results might be associated with using different parameters to compare with anti-RBD IgG: the previous study [18] used sVNT to compare with anti-RBD IgG, while our study used FRNT50 titer as a comparator. The result can be different due to a limitation of sVNT which does not measure all neutralizing antibodies but only the ones directed against the RBD, whereas the FRNT50 is a live virus neutralization test that more closely imitates the live virus infection than sVNT assay. Moreover, the different population studies might affect the inconsistent results. The study population of [18] was unvaccinated and vaccinated individuals with one or two doses of the vaccine, while our study population was individuals with booster vaccination which could elicit the broader neutralizing antibodies against SARS-CoV-2 variants, including the omicron variant [19].

In the comparison of omicron subvariants, although omicron BA.1 and BA.2 shared 12 amino acid alterations in RBD compared to wild-type D614G [20], the predicted anti-RBD IgG showed higher sensitivity and specificity to detect the neutralizing antibody for omicron BA.1 than for BA.2. For sVNT, the RBD recombinant protein was designed based on BA.1 omicron variants. As expected, the sensitivity and specificity between sVNT and BA.1 were higher than BA.2. In addition, our results show that the correlation analysis controlling for the groups of sex and age showed similar trends, suggesting that antibody response in individuals with booster vaccination might be less affected by sex and age differences [21].

There are several advantages to using anti-RBD IgG and sVNT to determine the antibody response against SARS-CoV-2. First, these methods do not require the live SARS-CoV-2 virus and a biosafety level 3 facility. Second, they do not require specially trained technicians and are suitable for use in hospitals and clinical laboratories. Additionally, these methods are used with high-throughput testing that is less time-consuming and takes 1–2 h to complete.

There are some limitations to this study. First, the sample size was relatively small. However, we addressed the performance analysis by using samples with a wide range of antibody concentrations. Second, we did not perform the sVNT against BA.2 due to the commercial recombinant RBD protein in the production process. Furthermore, the exact level of neutralizing antibodies that protect against SARS-CoV-2 infection has not yet been established.

In conclusion, the predicted anti-RBD IgG and sVNT levels corresponding to FRNT50 titers ≥ 20 against the omicron variant showed high sensitivity and specificity. This finding underscores that anti-RBD IgG and sVNT for the omicron variants can be used to predict the presence of neutralizing antibodies against omicron BA.1 and BA.2 subvariants.

## Figures and Tables

**Table 1:** Anti-RBD IgG against wild type, sVNT against omicron, FRNT50 titers against BA.1 and BA.2 among the booster vaccination groups.

Booster Groups		Anti-RBD IgG	sVNT	FRNT50 Titers BA.1	FRNT50 Titers BA.2
	*n*	GMT (95%CI)	Median (IQR)	GMT (95%CI)	GMT (95%CI)
**AZ + AZ + AZ**					
Pre-boost	20	67.4 (47.1–96.4)	NA	13 (10.4–16.2)	12.5 (10–15.6)
28 d post-boost	20	298.5 (204.2–436.2)	15 (4.8–21.7)	32.2 (20.1–51.6)	45.6 (28.8–72.3)
**AZ + AZ + HM**					
Pre-boost	20	46.8 (37.1–59)	NA	14.6 (11.6–18.5)	13 (10.4–16.1)
28 d post-boost	20	2160 (1649–2829)	65.7 (32–77)	396.5 (275.4–570.7)	224.5 (156.4–322.2)
90 d post-boost	20	901.2 (657–1236)	38 (22.5 -52.1)	119.1 (78.5–180.8)	110.5 (75–163)
**AZ + AZ + Mo**					
Pre-boost	20	43.6 (31.1–61.3)	NA	16.6 (13.2–21)	11 (9.6–12.6)
28 d post-boost	20	3034 (2418–3806)	67.7 (50.5–80.4)	547.8 (415.2–723)	324.2 (213.6–492.2)
90 d post-boost	20	916 (675–1243)	54.4 (35.5–88.9)	141 (89.6–221.6)	122 (71.4–208)
**AZ + AZ + PF**					
Pre-boost	20	43.3 (31.4–56.7)	NA	10 (10)	15 (11.8–19.1)
28 d post-boost	20	1876 (1581–2227)	70.3 (56.9–78)	166.3 (114–243.3)	247.7 (179.2–342.4)
90 d post-boost	20	556 (460–673)	32.5 (17.9–53.8)	78.1(47.5–128.2)	73.8 (56.1–97.2)
**SV + AZ + AZ**					
Pre-boost	10	146.5 (77.6–276.3)	11.48 (0.3–18.9)	12.8 (8.8–18.5)	24.4 (16.8–35.4)
28 d post-boost	20	315.8 (233.5–427)	11.4 (2.6–23.8)	40.3 (27.3–59.6)	59.3 (39.7–88.5)
**SV + AZ + Mo**					
Pre-boost	10	98 (66.2–145)	3.58 (1.0–6.6)	11 (8.9–13.5)	12 (9.1–15.7)
28 d post-boost	20	2930 (2156–3983)	79.7 (61.1–82.4)	271.6 (173–427)	235 (144–385.4)
**SV + AZ + PF**					
Pre-boost	10	135.4 (67.7–270.8)	8.1 (4.4–17.3)	17.3 (8.7–34.1)	28.3 (14.8–53.8)
28 d post-boost	20	3049 (2322–4005)	58.4 (33.1–78.5)	171 (120–243.3)	130.7 (78.9–216.8)

*SV* = *CoronaVac (Sinovac, China), AZ* = *AZD1222 (AstraZeneca, Oxford, UK), PF* = *BNT162b2 (Pfizer-BioNTech), Mo* = *full dose mRNA-1273 (100 µg) (Moderna), HM* = *half dose mRNA-1273 (50 µg).*

## Data Availability

All data are provided in the manuscript and Supplementary Files. Additional information can be requested from the corresponding author.

## Supplementary Materials

The following supporting information can be downloaded at: https://www.mdpi.com/article/10.3390/diagnostics12081781/s1. Table S1: Spearman’s R correlation analysis of anti-RBD IgG, surrogate virus neutralization test (sVNT), and FRNT50 titers against omicron BA.1 and BA.2; Figure S1: Spearman’s rank correlation between anti-RBD IgG and FRNT50 titers against omicron BA.1 determined controlling for the groups of age and sex; Figure S2: Spearman’s rank correlation between anti-RBD IgG and FRNT50 titers against omicron BA.2 analyzed controlling for the groups of age and sex; Figure S3: Spearman’s rank correlation between the percentage of inhibition measured by sVNT and FRNT50 titers against omicron BA.1 analyzed controlling for the groups of age and sex; Figure S4: Spearman’s rank correlation between the percentage of inhibition measured by sVNT and FRNT50 titers against omicron BA.2 analyzed controlling for the groups of age and sex.
