# Supplementary material for: Strong Correlations between the Binding Antibodies against Wild-Type and Neutralizing Antibodies against Omicron BA.1 and BA.2 Variants of SARS-CoV-2 in Individuals Following Booster (Third-Dose) Vaccination
Source: Diagnostics (Basel). 2022 Jul 22;12(8):1781. doi: 10.3390/diagnostics12081781 (PMC9394243; doi:10.3390/diagnostics12081781)
Supplement: Supplementary file 1 [file diagnostics-12-01781-s001.zip › Supplementary file/Supplementary file.pdf]

S1 Table Spearman's rank correlation analysis between anti-RBD IgG, the percentage of inhibition measured by surrogate virus neutralization test (sVNT) and FRNT50 titers against omicron BA.1 and BA.2

	n	Spearman's r	*p* -value
Anti-RBD IgG vs FRNT50 titers against omicron BA.1	310	0.89	*p*<0.001
Anti-RBD IgG vs FRNT50 titers against omicron BA.2	310	0.86	*p* <0.001
sVNT vs FRNT50 titers against omicron BA.1	218	0.77	*p* <0.001
sVNT vs FRNT50 titers against omicron BA.2	218	0.79	*p* <0.001

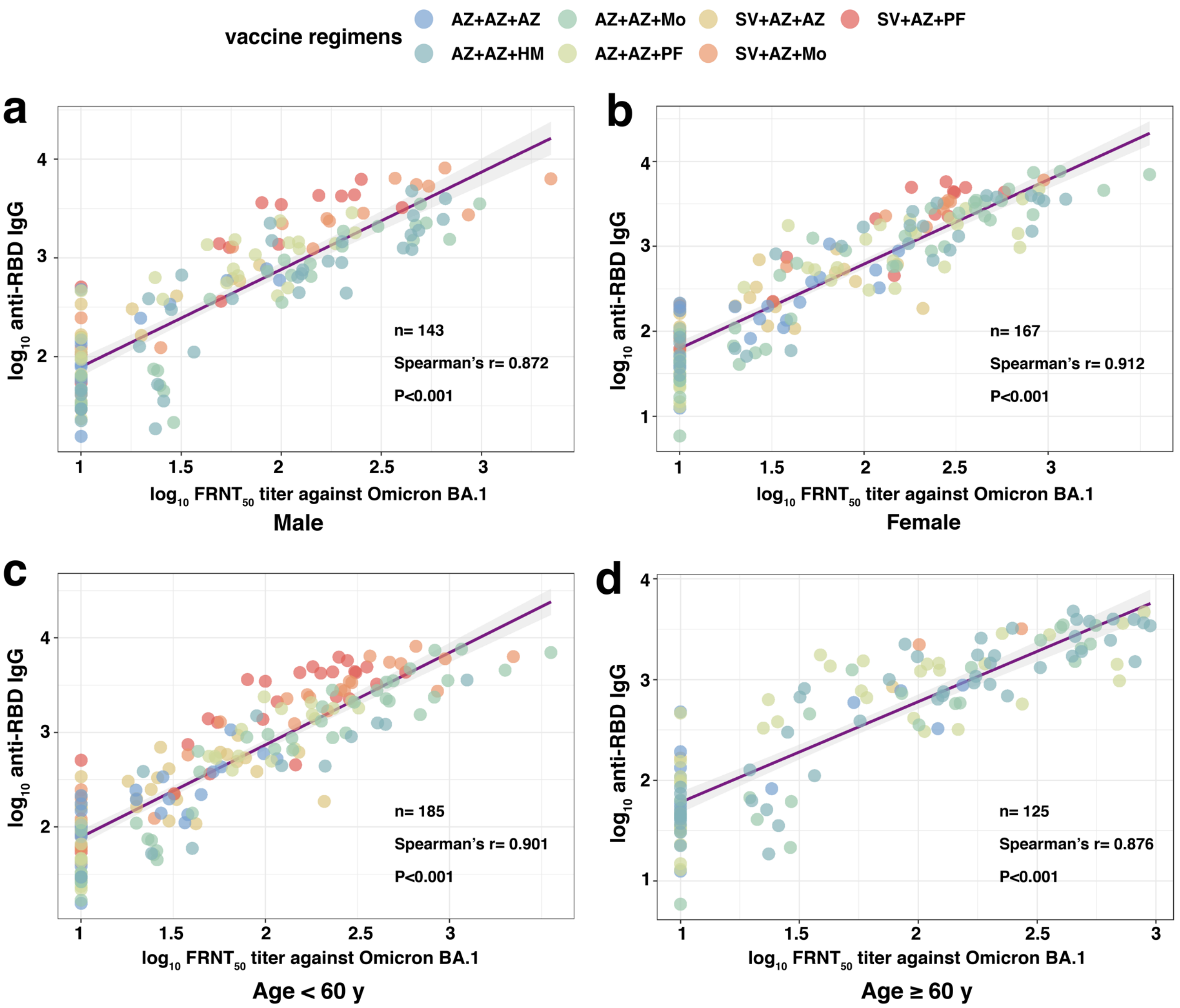

Figure S1 Spearman's rank correlation between anti-RBD IgG and FRNT50 titers against omicron BA.1 determined underlying the groups of age and sex. Individuals with booster vaccination were classified and analyzed by groups of sex, (a) male and (b) female, by ages (c) age <60 years old and (d) age ≥ 60 years old. FRNT50, foci reduction neutralization test 50; RBD, receptor-binding domain.

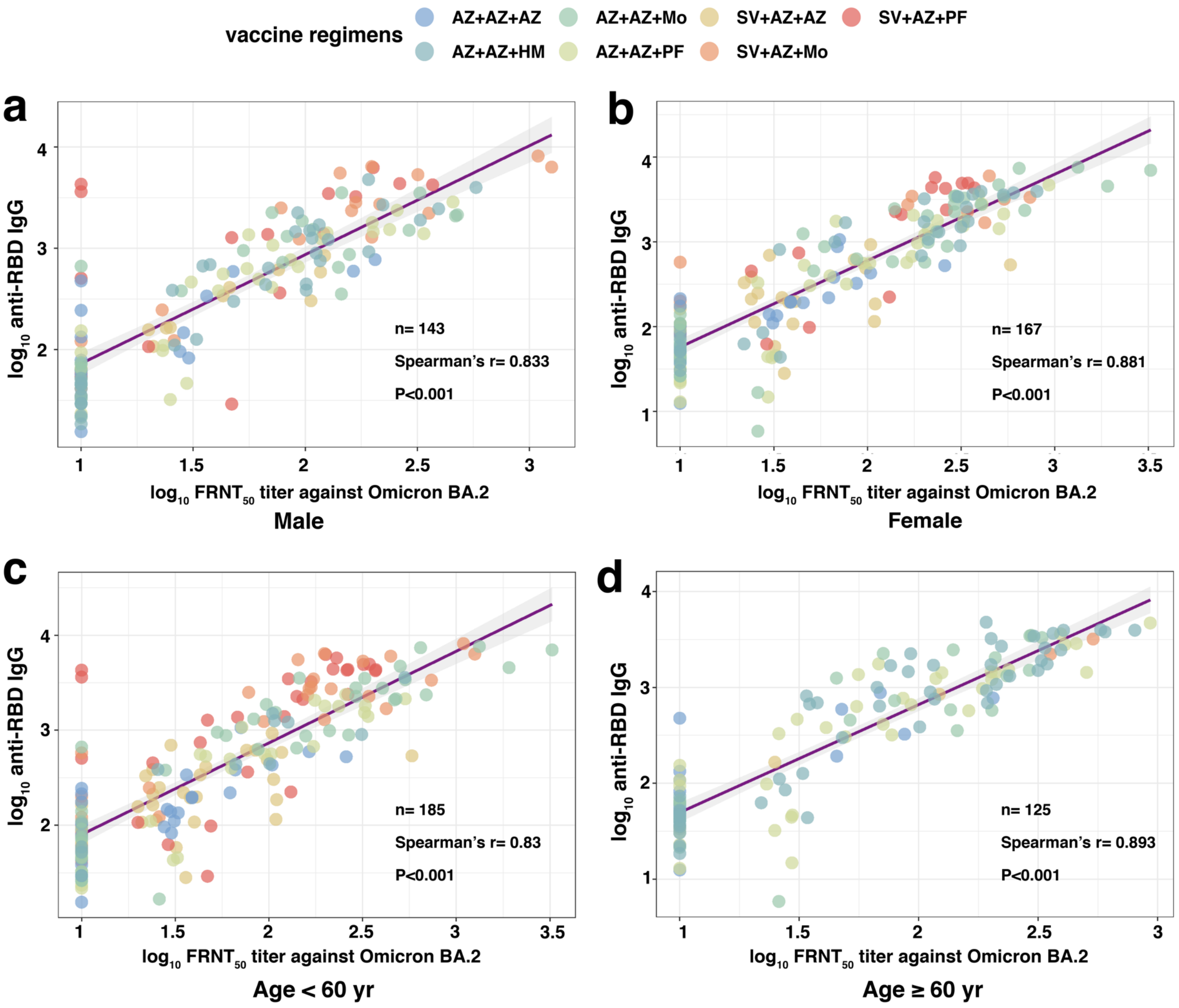

Figure S2 Spearman's rank correlation between anti-RBD IgG and FRNT50 titers against omicron BA.2 determined underlying the groups of age and sex. Individuals with booster vaccination were classified and analyzed by groups of sex, (a) male and (b) female, by ages (c) age <60 years old and (d) age ≥ 60 years old. FRNT50, foci reduction neutralization test 50; RBD, receptor-binding domain.

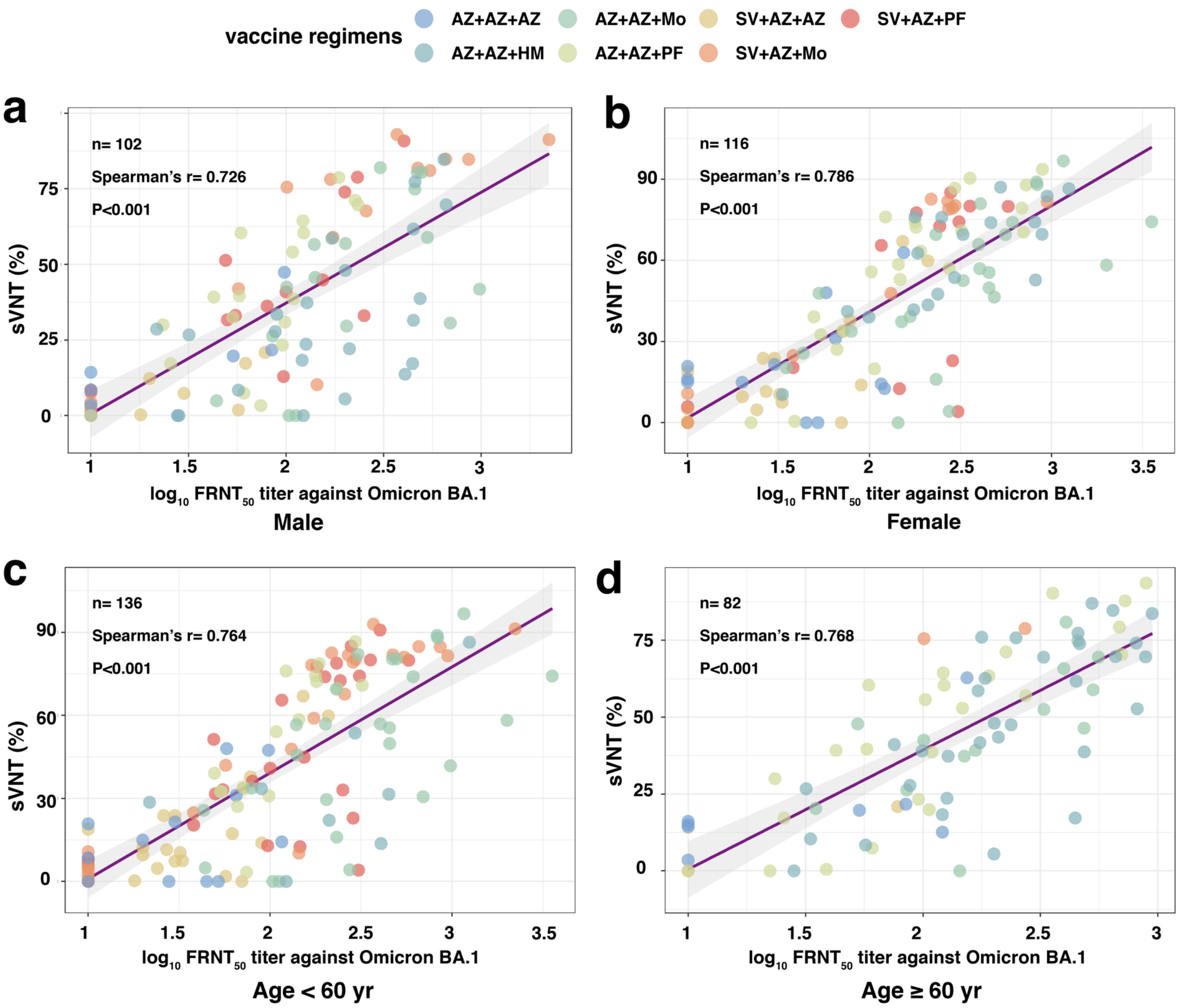

Figure S3 Spearman's rank correlation between the percentage of inhibition measured by sVNT and FRNT50 titers against omicron BA.1 determined underlying the groups of age and sex. Individuals with booster vaccination were classified and analyzed by groups of sex, (a) male and (b) female, by ages (c) age <60 years old and (d) age ≥ 60 years old. FRNT50, foci reduction neutralization test 50; sVNT, surrogate virus neutralization test.

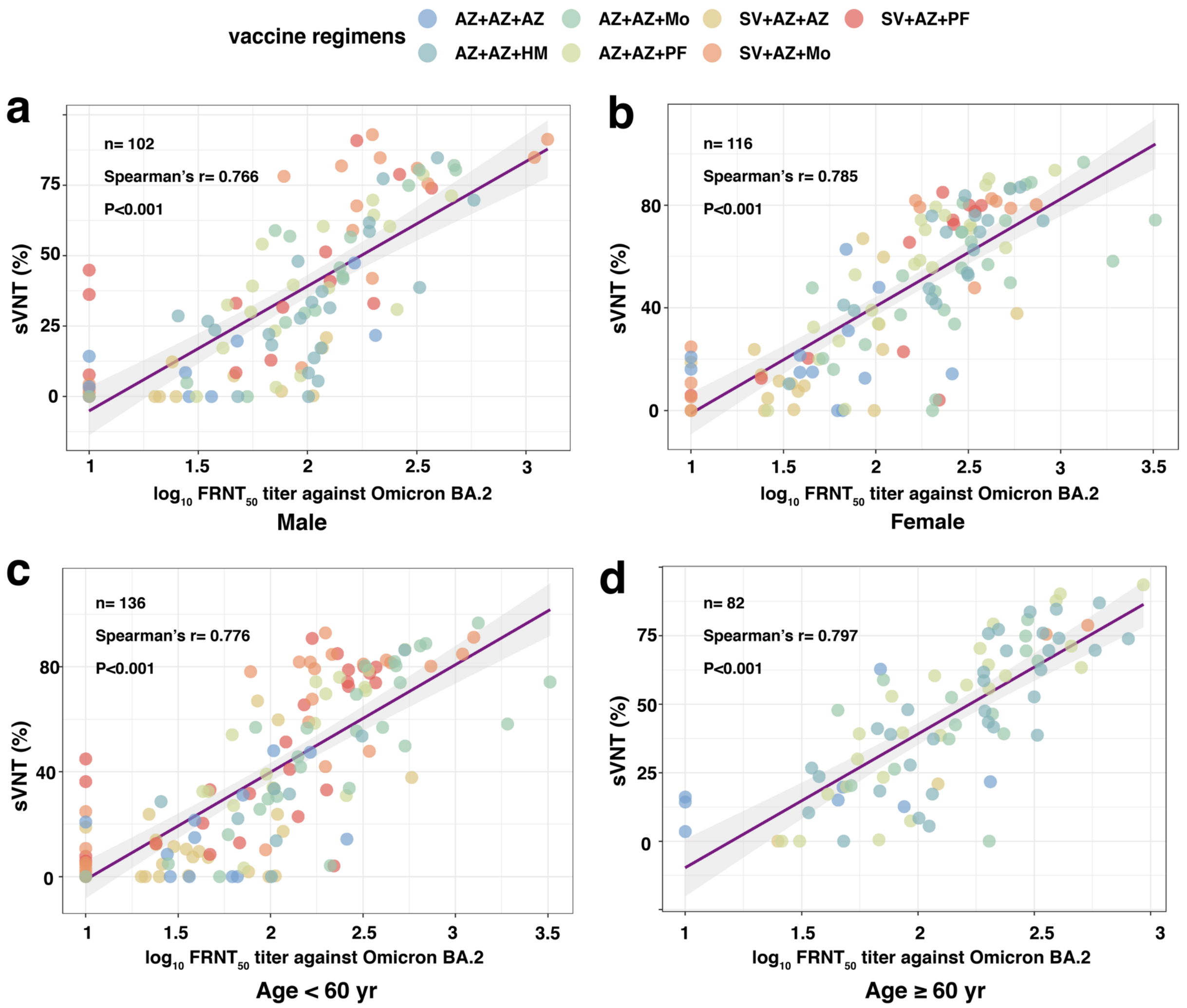

Figure S4 Spearman's rank correlation between the percentage of inhibition measured by sVNT and FRNT50 titers against omicron BA.2 determined underlying the groups of age and sex. Individuals with booster vaccination were classified and analyzed by groups of sex, (a) male and (b) female, by ages (c) age <60 years old and (d) age ≥ 60 years old. FRNT50, foci reduction neutralization test 50; sVNT, surrogate virus neutralization test.
